# Who is utilizing anti-retroviral therapy in Ghana: An analysis of ART service utilization

**DOI:** 10.1186/1475-9276-11-62

**Published:** 2012-10-16

**Authors:** Phyllis Dako-Gyeke, Rachel Snow, Alfred E Yawson

**Affiliations:** 1 Department of Social and Behavioral Sciences, School of Public Health, College of Health Sciences, University of Ghana, P. O. Box LG 13, Accra, Ghana; 2 Department of Health Behavior and Health Education, School of Public Health, University of Michigan, 109 Observatory, 3814, Ann Arbor, MI, 48109-2029, USA; 3 Department of Community Health, University of Ghana Medical School, College of Health Sciences, P. O. Box 4236, Korle-Bu, Accra, Ghana; 4 National AIDS/STIs Control Program, Ghana Health Service, Korle- Bu, Accra, Ghana

**Keywords:** Inequities, ART, Utilization, Sex, Regional, Ghana, HIV and AIDS

## Abstract

**Introduction:**

The global scale-up of antiretroviral therapy (ART) for HIV patients has led to concerns regarding inequities in utilization of ART services in resource-limited contexts. In this paper, we describe regional and sex differentials in the distribution of ART among adult HIV patients in Ghana. We highlight the need for interventions to address the gender-based and geographic inequities related to the utilization of ART services in Ghana.

**Methods:**

We reviewed National AIDS/STIs Control Program’s ART service provision records from January 2003 through December 2010, extracting data on adults aged 15+ who initiated ART in Ghana over a period of eight years. Data on the number of patients on treatment, year of enrollment, sex, and region were obtained and compared.

**Results:**

The number of HIV patients receiving ART in Ghana increased more than 200-fold from 197 in 2003, to over 45,000 in 2010. However, for each of six continuous years (2005-2010) males comprised approximately one-third of adults newly enrolled on ART. As ART coverage has expanded in Ghana, the proportion of males receiving ART declined from 41.7% in 2004 to 30.1% in 2008 and to 27.6% in 2010. Also, there is disproportionate regional ART utilization across the country. Some regions report ART enrollment lower than their percent share of number of HIV infected persons in the country.

**Conclusions:**

Attention to the comparatively fewer males initiating ART, as well as disproportionate regional ART utilization is urgently needed. All forms of gender-based inequities in relation to HIV care must be addressed in order for Ghana to realize successful outcomes at the population level. Policy makers in Ghana and elsewhere need to understand how gender-based health inequities in relation to HIV care affect both men and women and begin to design appropriate interventions.

## Introduction

The global expansion of antiretroviral therapy (ART) for HIV patients has raised concerns regarding inequities in utilization of ART services within countries [1-4]. The Global Fund, PEPFAR, WHOs “3 by 5” initiative, and efforts by other public and private organizations have ensured the scale-up of ART in resource-constrained settings over the last 8 years [5-8]. In 2010 it was estimated that approximately 6.6 million people in low- and middle-income countries were receiving HIV treatment, representing approximately 47% of people who needed treatment [9]. In Ghana, a total of 33,745 people were receiving ART by the end of 2009 [10]. The availability of ART has transformed what was once a deadly disease into a manageable chronic condition [1]. However due to geographical and gender-based health inequities related to HIV treatment, not all patients in need of ART in resource-limited settings utilize the service.

Inherent challenges such as limited institutional, financial, and human resources can cause geographic disparities in utilization of ART within resource-limited contexts [11,12]. Additionally, gender-based inequities have been increasingly identified with HIV-related care and treatment, as well as general health care utilization [13,14]. Theoretical work on why males and females differ in health seeking behavior has seen extensive, if not decisive, treatment in the global health and sociological literature [15-18]. This literature suggests that gender inequities, norms and attitudes may work differently in different contexts to hinder either men or women from accessing HIV treatment [13,14,19-22]. Even though women are often more susceptible to HIV infection than men [23], their access to HIV treatment can be constrained by limits on their mobility, limited control over household resources, poverty, lack of health insurance, social support, or fear of stigma [13,21-25]. Although men’s utilization of ART can be inhibited for similar reasons, [26-28] compelling arguments have been made that hegemonic masculinities across many cultures include demands that men under-attend to potential physical weaknesses [16,17,29]. Consequently, men are keen to identify as ‘not feminine’, which includes avoiding frequent and trivial use of health services [18]. We would argue that such anxieties may be augmented for the diagnosis and treatment of HIV or other potential sexual infections, in which the patient-provider interaction must inevitability focus on issues of sexual ill-health, and potential fears of sexual disempowerment.

Several sex disaggregated analyses conducted in various parts of Africa show either high proportions of females receiving ART in some countries (South Africa, Botswana, Ethiopia, Ivory Coast, Malawi, and Zimbabwe) [13,14,30-32] or high male utilization of ART in others (Swaziland, and Zambia) [14]. Advancing equity in health means addressing gender-based and geographical disparities associated with the utilization of ART services across Africa.

In Ghana, the HIV population is estimated at approximately 221,941 [33], and the HIV virus has spread to all ten administrative regions in the country. ART services have been available to HIV patients since June 2003; guidelines from the National AIDS/STIs Control Program (NACP) recommend patients to initiate treatment when their CD4 count is less than 350 cells/ml and/or they become symptomatic with HIV infection in WHO stage III or IV [34]. Although there has been an increase in the number of patients receiving ART, approximately 70% of the HIV- infected population in Ghana is not on treatment [35,36]. UNAIDS identified Ghana as one of 11 countries with less than 40% ART coverage in 2009 [36]. To help address this problem, the Ghanaian government is currently making efforts to increase coverage to 60% of those eligible for treatment by 2013 [10]. To inform the further scale up of ART services, we undertook a review of patients receiving ART in the country. Both the regional and sex distribution of patients receiving ART, especially when cost of ART no longer seems to be a constraint, are highlighted.

Rigorous examination of current ART programs in resource-limited settings is critical to identify implementation gaps and to suggest context-specific interventions that can improve accessibility for all eligible HIV patients [1,37]. It is imperative to address inequities regarding access and utilization of ART, ensuring wide spread and timely access, as well as prolonged healthy living among HIV patients [6]. Prolonged healthy living helps HIV response efforts at the population level by reducing stigma, promoting prevention-oriented behavior and thereby reducing the further spread of the virus [9]. To document the regional and sex disparities, this study reviews NACP dataset [10,38].

## Methods

The data source for this review is NACP sex and region disaggregated records on adults aged 15+ who initiated ART from 2003 to 2010. The NACP is responsible for coordination and implementation of HIV and AIDS-related aspects of the Ghana Health Strategic Framework. Implementation is managed by the Disease Control and Prevention Department of the Public Health Directorate of the Ghana Health Service. NACP sources and collates computerized HIV and AIDS- related service provision data from community health centers, district hospitals, regional hospitals and teaching hospitals throughout the country, every quarter. From these records, NACP generates a comprehensive national dataset that covers service provision across all ten administrative regions in Ghana. These data were the source for the 2010 Ghana’s Progress Report on the United Nations General Assembly Special Session [10].

National and regional HIV prevalence for adults aged 15-49 years are estimated by NACP from antenatal and STI surveillance data, using recommended United Nations’ algorithms [39]. Regional adult population figures provided in Table 1 were generated using the United States Census Bureau’s 2010 estimation that 52% of the Ghanaian population is aged between 15-49 years [40]; this was applied to the 2010 Ghana population and housing census data for each region [41]. In order to determine who is utilizing ART, the sex of adult HIV patients receiving ART in each year (2003-2010) are displayed. Also, trends are examined by comparing new ART enrollments (sex and total) over time. In addition, a detailed analysis of the geographic (regional) location of patients receiving treatment in the year 2010 is provided by contrasting the proportion of the total Ghanaian population residing in each region with the proportion of the total HIV + population, and the ART-using population in that region; discrepancies are noted. Descriptive statistics include simple frequencies, proportions, percentages and ratios. These are presented by year of enrollment, sex and region. All analyses were performed using the statistical software package SPSS version 19.

**Table 1:** Regional Distribution of HIV Patients on ART

**Regional Distribution of HIV Patients Enrolled on ART, By Population Distribution & HIV Prevalence (2010)**						
**Region**	**Adult Population Share^a^**	**Adult HIV Prevalence, 2010^b^**	**Number of Adults Infected^c^**	**Regional Share of Ghana’s Infected Adults**	**Number Started on ART, 2010**	**Regional Share of Adults who Initiated ART in Ghana, in 2010**
Ashanti	19.5% (2457024)	3.0%	73711	25.4%	2395	20.2%
Eastern	10.7% (1349927)	3.2%	43198	14.9%	2116	17.8%
Greater Accra	16.1% (2033077)	2.6%	52860	18.2%	1952	16.5%
Brong Ahafo	9.4% (1186707)	2.0%	23734	8.2%	1432	12.1%
Volta	8.7% (1091936)	1.8%	19655	6.8%	1118	9.4%
Western	9.6% (1209310)	2.5%	30233	10.4%	1,048	8.8%
Central	8.7% (1095749)	1.7%	18628	6.4%	609	5.1%
Northern	10.2% (1283650)	0.7%	8986	3.1%	464	3.9%
Upper West	2.8% (352437)	1.7%	5991	2.1%	347	2.9%
Upper East	4.3% (536369)	2.4%	12873	4.4%	385**	3.2%
Total	100% (12596184)		289868	100.0%	11866	100.0%

** 2009 data for Upper East was used due to missing data for 2010.

^a^Adult population (15-49 years) share estimated as 52% of total population for each region.^33,34^

^b^Regional adult HIV prevalence.^32^

^c^Number infected is estimated using adult HIV prevalence and adult population share.

## Results

### ART utilization trends in Ghana

**Figure 1:**
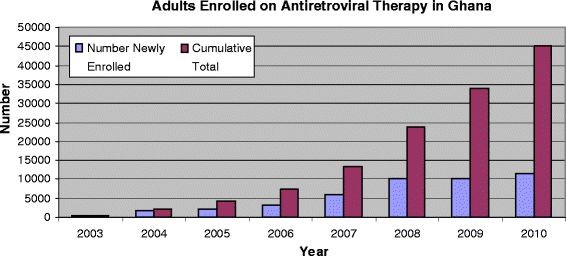
**Adults Enrolled on ART in Ghana (2003-2010).** Number of adults enrolled on antiretroviral therapy in Ghana from 2003 to 2010. Figure shows total number of adult HIV patients in Ghana who newly enrolled on antiretroviral therapy each year starting from 2003 to 2010. Figure also demonstrates the cumulative total of adult HIV patients who enrolled on antiretroviral therapy across the country starting from 2003 to 2010.

The data (Figure 1) show- high increases in the number of adults enrolled on ART from 2003 to 2004, and again from 2006 to 2007. When ART was initially rolled-out in 2003, 197 adults enrolled for the service. In the second year (2004) new enrollees increased to almost 2,000 adults, representing a 900% increase in one year. In 2007 a record 6,091 adults enrolled, an approximately two-fold increase in enrollments the previous year (Figure 1). By the end of 2010 the cumulative number of adults enrolled on ART in Ghana was approximately 45,000.

### Regional distribution of HIV patients on ART in Ghana

Regional HIV prevalence rate in 2010 range from 3.2% in the Eastern region to 0.7% in the Northern region (Table 1). With a 3.0% HIV prevalence rate, Ashanti region contributed approximately 25.4% of the total number of HIV infected persons in Ghana, in the year 2010. Ashanti, Eastern and Greater Accra regions each accounted for a greater share in total HIV-positive cases in Ghana than their share of the overall adult population in the country. This discrepancy was greatest in the Eastern region, which represents 10.7% of the adult Ghanaian population, but 14.9% (*i.e.* 39% more) of the adult HIV-positive cases in Ghana. The remaining seven regions of Ghana had proportions of HIV-positive cases that were either comparable or less than their share of the adult population.

The Ashanti region had the highest share of ART enrollees (20.2%) in the year 2010 (Table 1), notably less than their 25% share of HIV-positive adults. Greater Accra also had proportionately more HIV-positive cases than they had enrollees on ART (Table 1). At the same time, the Eastern, Brong Ahafo and Volta regions had proportionately more adults using ART than their share of HIV-positive persons. Other regions where the percent share of overall ART enrollments was lower than the region’s percent share of HIV infections included Western, Central and Upper East) regions (Table 1).

### Sex differentials in the distribution of patients on ART

**Figure 2:**
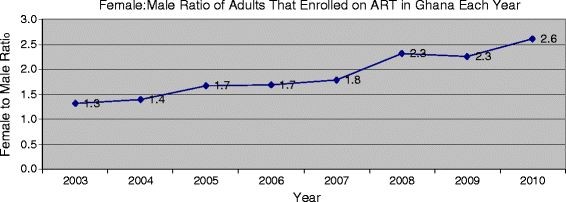
**Female to Male Ratio of Adults Enrolled on ART in Ghana.** Figure shows female to male ratio of adults enrolled on antiretroviral therapy in Ghana for each year, starting from 2003 to 2010. Figure shows the female to male ratio of total number of adult HIV patients in Ghana who newly enrolled on antiretroviral therapy each year starting from 2003 to 2010.

For the 8 year period within which ART services have been available in Ghana, more females than males have been enrolling (Table 2). In 2003 43.1% of adults enrolled on ART were males, and this proportion declined precipitously in the following years, progressing to 41.3% in 2004, to 30.1% in 2008 and 27.6% in 2010 (Table 2). The F:M ratio was 1.3 in 2003, but steadily increased over the years (Figure 2).

**Table 2:** Proportion of Females and Males on ART

**Proportion of Females and Males Enrolled on ART From 2003 - 2010**					
**Year**	**Total Number of Patients Enrolled**	**FEMALES**		**MALES**	
		**Number Enrolled**	**Proportion**	**Number Enrolled**	**Proportion**
2003	197	112	0.569	85	0.431
2004	1831	1067	0.583	764	0.417
2005	2032	1270	0.625	762	0.375
2006	3278	2060	0.628	1218	0.372
2007	6091	3911	0.642	2180	0.358
2008	10185	7119	0.699	3066	0.301
2009	10131	7027	0.694	3104	0.306
2010	11481	8315	0.724	3166	0.276

### Sex distribution of HIV patients on ART in the ten regions of Ghana

In the most recent year of data (2010), the F:M ratio in all ten regions was above 2.0 (Table 3). The Central region recorded the highest F:M ratio (3.4), followed by three regions (Brong Ahafo, Volta, and Upper East) each recording 2.9 F:M ratio in 2010 (Table 3). The lowest F:M ratio of 2.4 was reported by Ashanti, Eastern and Upper West Regions (Table 3).

**Table 3:** Enrollment of Patients on ART

**Enrollment of Patients on ART in All Regions in 2010 by Sex; Showing F: M Ratio**			
**Region**	**Total Number Started on ART in 2010**	**Number of Males Started on ART in 2010**	**F:M Ratio**
Ashanti	2395	702	2.4
Eastern	2116	624	2.4
Greater Accra	1952	536	2.6
Brong Ahafo	1432	364	2.9
Volta	1118	285	2.9
Western	1048	286	2.7
Central	609	138	3.4
Northern	464	130	2.6
Upper West	347	101	2.4
Upper East	385**	100	2.9
Total	11866	3266	2.6

** 2009 data for Upper East was used due to missing data for 2010.

## Discussion

Despite profound increases in ART utilization, lower proportions of males are obtaining ART services in Ghana. For each of the six recent years (2005-2010), males consisted of less than one third of adults who newly enrolled on ART each year. The proportion of males who newly enrolled declined from 41.7% in 2004 to 27.6% in 2010. Our finding is consistent with Family Health International’s report on pilot projects that initiated ART in Ghana, Kenya and Rwanda [42]. The FHI report indicates women comprised a majority (62%) of ART patients in all three countries [42]. Also, several studies conducted in other parts of Africa (South Africa, Botswana, Ethiopia, Ivory Coast, Malawi, and Zimbabwe) indicate similar over-representation of women in persons receiving ART [13,14,30-32]. After conducting a systematic review, Muula and his colleagues found a F:M ratio of patients receiving ART to be equal to, or greater than 1, in all but 2 of 21 published studies in seven Southern African countries [14].

The over-representation of women receiving ART may partially reflect the sex proportions of the adult population infected with HIV in African countries [43]. For instance, the 2003 Ghana Demographic and Health Survey reports nearly 3% national HIV prevalence for women aged 15-49 years, but less than 2% for men aged 15-59 years [44], suggesting an F:M ratio of 1.8 to 1. Also, the Ghana NACP 2010 [33] HIV estimates suggest that the F: M ratio of the HIV-positive population in Ghana is 1.3:1. Irrespective of the comparatively high HIV prevalence among females, the Ghana AIDS Commission claims that in 2009, 50% of females who needed ART were accessing the service while only 39% of males who needed ART had access [10], suggesting a 1.2:1 F:M ratio.

Wagner and his colleagues argue that most people do not seek HIV testing and care until they are experiencing severe symptoms and thus have a more advanced disease stage [7]. This situation is more likely the case for males in Africa as studies indicate that men start treatment with more advanced stages of illness which negatively impacts their prognosis [13,45]. In investigating the relationship between patient’s clinical stage and CD4 count in Ghana, Torpey and his colleagues observed that seeking testing and care at a later HIV stage, and with very low CD4 count jeopardizes treatment outcomes [46].

The underrepresentation of males in ART enrollment may be attributed to lower HIV testing among males compared to females. Several studies in sub-Saharan Africa suggest women test for HIV more than men [44,47,48] and also start treatment at an earlier clinical stage of HIV than men [13]. Since HIV testing serves as the funnel into HIV care and eventual ART initiation the underrepresentation of males in ART utilization in Ghana may simply be a direct consequence of the documented low HIV testing among men [7,44,47-49]. A recent four-year review (2007-2010) of HIV Testing and Counseling data from the NACP in Ghana found that F:M ratio of HIV testing ranged from 7.6 in 2007 to 4.2 in 2010 [49]. This finding was also confirmed by the 2008 Ghana Demographic and Health Survey which reported that women (17%) were slightly more likely to have been tested for HIV and received their results than men (12%) [48].

More structural and pragmatic explanations for male under-utilization of both HIV testing and ART enrollments are plausible, including the fact that women are generally more acculturated to the routine use of health services through child-bearing and responsibility for children’s health. This may be particularly salient in African contexts where annual physical check-ups or health screening services are not yet routine for adult men or women. In this context, improvements in access to HIV testing among women attending antenatal care has been noted as a key entry point for women into HIV- related care [14]. In Ghana, HIV testing among pregnant women is encouraged as part of efforts to prevent mother to child transmissions.

Furthermore, researchers on masculinity and health generally attribute low male utilization of health care services to traditional concepts of masculinity that disassociate male identity from disease and project males as invulnerable and not needing health care [16,17,29].

We would argue that such anxieties may be augmented for the diagnosis and treatment of HIV or other potential sexual infections, in which the patient-provider interaction must inevitability focus on issues of sexual ill-health, and potential fears of sexual disempowerment. That said, one should adopt western theories to the Ghanaian context with some degree of caution, given evidence of wide-ranging strategies for affirming dominant masculinity across social class, profession, or cultural location even within the USA or UK [50,51]. Instead, further investigation is warranted in the Ghanaian setting.

In addition to wide sex differentials, there is disproportionate ART use across the ten administrative regions in the country. Ashanti region, being the most populous region in the country, had the highest ART enrollments, followed by Greater Accra and Eastern regions. Considering that Accra, the capital of Ghana is located within the Greater Accra region, it is possible that the provision of ART in this region has become more feasible, partly due to the wide availability of health facilities within the urban sites of the region. Also the Eastern region which consistently records the highest HIV prevalence in the country continues to receive attention. Therefore, the high ART enrollment in the Eastern region may reflect interventions aimed at addressing the high HIV prevalence in that region.

Besides these three regions, other regions report ART enrollment that is either lower or higher than the region’s percent share of number of HIV infected persons in the country. For instance, Brong Ahafo and Volta regions had comparatively high shares of ART enrollment, 12.1% and 9.4%, respectively. These are more than their respective percent shares of number of HIV infected persons (Brong Ahafo, 8.2%; Volta, 6.8%). The regional utilization patterns demonstrate how scale-up efforts occurring simultaneously within different African settings may, or may not be necessarily responding to specific geographical needs [7]. For Ghana, these estimates highlight the regional differences in population HIV-burdens, and reflect the different coverage of ART for HIV-positive persons across Ghana.

Regardless of sex and regional differentials, there is an upward trend in ART utilization across the country. Although few HIV patients (197) enrolled when ART was initially piloted in June 2003 in Ghana, this effort laid the foundation for expansion of ART services in subsequent years. The records show over a two-hundred-fold increase in the number of HIV patients on ART across the country by the end of 2010. Similar increasing ART utilization trends were found in various resource-limited settings during this period [12,52,53]. One important factor that accounts for this steady increase in ART utilization in Ghana is the concurrent increase in the number of sites providing ART services. According to national reports, facilities providing ART services in Ghana increased from 3 in 2003, to 13 sites in 2005 [10]. By December 2009, this number had profoundly increased to 138. These are health facilities that provide ART for HIV patients at the district, regional and (Tertiary) national health facilities in both the public and private sector [10]. The enormous increase in number of adults enrolled on ART each year is indicative of numerous ART scale-up efforts initiated by public and private organizations across Africa including governments, non- government, and community-based organizations [7]. In Ghana, the scale-up has continued in the public sector with linkages to the private sector through the NACP [10].

Whereas the upward trend in ART utilization across the country provides evidence of the wide scale-up efforts, sex and regional disparities in utilization highlight HIV-related healthcare inequities that need to be further investigated and addressed. Although inherent challenges such as limited institutional resources and service capacity, may explain such gaps, the availability of a wide spectrum of alternative services for HIV patients in Ghana may explain why some HIV patients do not utilize ART services. Research conducted by Awusabo-Asare and Anarfi on the health-seeking behavior of persons with HIV and AIDS in Ghana indicate that, after being diagnosed, HIV patients in Ghana may also visit the traditional healer or spiritualist considering the supernatural explanations given to HIV infection in some cases [54]. Also, other studies suggest the possibility of HIV patients seeking care through the informal sector [13]. Preference for the informal sector may be due to fear of stigma and disclosure of HIV status, perception that hospitals are unfriendly and confusing, and work or family responsibilities [6,13]. Given that these factors may unequally affect women and men, as well as the various regions in Ghana, understanding how and why these impact the health seeking behavior of each gender and each region warrants more inquiry. It will be important to investigate if indeed, with the availability of ART, male patients, vis-à-vis female patients, are more likely to engage the services of alternative health care outlets. Also, differences between the ten administrative regions in the proportion of persons likely to solicit HIV-related care from the informal sector warrant investigation.

Furthermore, disparities in utilization of ART can be explained by inequities related to access to health service benefits in Ghana. Studies have shown differentials in the distribution of health service benefits across various subgroups in Ghana [55]. For instance, geographical access to health care services has been identified as a key challenge for rural populations in Ghana [55]. Unfortunately, such barriers potentially prevent some eligible HIV patients from having equal and timely access to treatment, thereby further widening the health inequity gap. In this context, promoting equal access to ART means addressing inherent inequities in access to health service benefits in the country. Structures must be put in place to ensure timely access to ART by all eligible HIV patients irrespective of gender, age, location or socio-economic status.

In this study, we reviewed a national dataset to describe the sex and regional distribution of HIV patients receiving ART in Ghana. The limitation, however, is that this dataset is based on routine service provision records that may sometimes be incomplete. Also, since these data do not include other demographic information on patients it is difficult to determine double counting or to identify patients who are no longer receiving the service due to death or loss to follow-up. Another limitation is in using the national proportion of the 15-49 year age group to estimate the regional adult population. It is important to note, however, that the US Census Bureau estimations, the source for these regional estimations, uses the Ghana Demographic Health Survey, a population-based survey which provides good coverage of the general population [56]. The GDHS is also used in calculating population estimations of HIV prevalence in Ghana [39,57]. Despite these limitations, it is remarkable to note that our study uses data from different time periods and different regions within the country to show the disproportionally lower use of ART services by males. Also, by using a national dataset we were able to look critically at the outcome of the ART scale-up efforts in the launching years from 2003 to 2010, offering insight into operational realities.

## Conclusions

Considering findings from this study and several others, it is clear that men are underrepresented in the distribution of HIV patients receiving ART in Ghana, and several other African settings. Unfortunately, this has led to increased mortality among the male HIV- population in Africa [13,45]. Although explanations for increased mortality among men include men’s poor healthcare decisions, service structures need to enhance their capacity to attract and serve males [20]. Policy makers in Ghana and elsewhere need to understand how gender inequality affects both men and women and begin to design gender-based interventions. Barriers against accessing HIV-related care amongst men must be investigated. In addition, HIV- related services, such as HIV testing, can be established within male-dominated spaces, *e.g.* in locales such as sports arenas or social clubs. By so doing HIV-related care will be brought closer to males. ART scale-up efforts across Africa should pay candid attention to sex and regional disparities if indeed we want to encourage high involvement in HIV-related care by all eligible subgroups.

## Abbreviations

ART: antiretroviral therapy; PEPFAR: President’s Emergency Plan for AIDS Relief; WHO: World Health Organization; NACP: National AIDS/STIs Control Program; GHS: Ghana Health Service.

## Competing interests

The authors declare that they have no competing interests.

## Authors’ contributions

All authors (PDG, RC and AEY) contributed to the conception, analysis and interpretation of data. AEY assisted with acquisition of the dataset. PDG, RC and AEY participated in the writing of the manuscript. All authors read and approved the final manuscript.
